# Assessment of neuropharmacological potential of low molecular weight components extracted from *Rhinella schneideri* toad poison

**DOI:** 10.1590/1678-9199-JVATITD-1484-18

**Published:** 2019-04-18

**Authors:** Mateus Amaral Baldo, Alexandra Olimpio Siqueira Cunha, Lívea Dornela Godoy, José Luiz Liberato, Juliana Sakamoto Yoneda, Elisa Correa Fornari-Baldo, Pietro Ciancaglini, Wagner Ferreira dos Santos, Eliane Candiani Arantes

**Affiliations:** 1 Department of Physics and Chemistry, Ribeirão Preto College of Pharmaceutical Science, University of São Paulo, Ribeirão Preto, SP, Brazil; 2 Neurobiology and Venoms Laboratory, Department of Biology, Faculty of Philosophy, Sciences and Letters at Ribeirão Preto, University of São Paulo, Ribeirão Preto, SP, Brazil; 3 Institute of Neurosciences & Behavior - INeC, Campus USP, Ribeirão Preto, SP, Brazil; 4 Health and Science Institute, Paulista University, São Paulo, SP, Brazil; 5 Department of Chemistry, Faculty of Philosophy, Sciences and Letters at Ribeirão Preto, University of São Paulo, Ribeirão Preto, SP, Brazil

**Keywords:** Rhinella schneideri, toad poison, bufadienolides, seizures, neuroprotection

## Abstract

**Background:**

Studies on toad poison are relevant since they are considered a good source of toxins that act on different biological systems. Among the molecules found in the toad poison, it can be highlighted the cardiotonic heterosides, which have a known mechanism that inhibit Na^+^/K^+^-ATPase enzyme. However, these poisons have many other molecules that may have important biological actions. Therefore, this work evaluated the action of the low molecular weight components from *Rhinella schneideri* toad poison on Na^+^/K^+^-ATPase and their anticonvulsive and / or neurotoxic effects, in order to detect molecules with actions of biotechnological interest.

**Methods:**

*Rhinella schneideri* toad (male and female) poison was collected by pressuring their parotoid glands and immediately dried and stored at -20 °C. The poison was dialysed and the water containing the low molecular mass molecules (< 8 kDa) that permeate the dialysis membrane was collected, frozen and lyophilized, resulting in the sample used in the assays, named low molecular weight fraction (LMWF). Na^+^/K^+^ ATPase was isolated from rabbit kidneys and enzyme activity assays performed by the quantification of phosphate released due to enzyme activity in the presence of LMWF (1.0; 10; 50 and 100 µg/mL) from *Rhinella schneideri* poison. Evaluation of the L-Glutamate (L-Glu) excitatory amino acid uptake in brain-cortical synaptosomes of Wistar rats was performed using [3H]L-glutamate and different concentration of LMWF (10^-5^ to 10 µg/µL). Anticonvulsant assays were performed using pentylenetetrazole (PTZ) and N-methyl-D-aspartate (NMDA) to induce seizures in Wistar rats (n= 6), which were cannulated in the lateral ventricle and treated with different concentration of LMWF (0.25; 0.5; 1.0; 2.0; 3.0 and 4.0 µg/µL) 15 min prior to the injection of the seizure agent.

**Results:**

LMWF induced a concentration-dependent inhibition of Na^+^/K^+^-ATPase (IC_50%_ = 107.5 μg/mL). The poison induces an increased uptake of the amino acid L-glutamate in brain-cortical synaptosomes of Wistar rats. This increase in the L-glutamate uptake was observed mainly at the lowest concentrations tested (10^-5^ to 10^-2^ µg/µL). In addition, this fraction showed a very relevant central neuroprotection on seizures induced by PTZ and NMDA.

**Conclusions:**

LMWF from *Rhinella schneideri* poison has low molecular weight compounds, which were able to inhibit Na^+^/K^+^-ATPase activity, increase the L-glutamate uptake and reduced seizures induced by PTZ and NMDA**.** These results showed that LMWF is a rich source of components with biological functions of high medical and scientific interest.

## Background

Toxins and animal poisons as well as the molecules that are synthesized by plants are considered natural products and have emerged throughout the evolutionary process due to adaptation in various livable environments. These molecules generally interact with specific targets and, because of this interaction, they are capable of inducing pharmacological or toxicological effects [1, 2].

*Rhinella schneideri (B. schneideri Werner,* 1894*)* is a toad belonging to Bufonidae family and popularly known as true toad [3, 4]. These animals showed amendments in their skin, which allowed the adaptation in the terrestrial environment. These modifications came in their skin with glands that produce a wide variety of molecules that enable to defend themselves against pathogenic agents and predators [5, 6].

Among the molecules that were found in the toad poison, it can be mentioned bufadienolides, peptides, alkaloids and biogenic amines [7-11].

The crude toad poison, as well as isolated molecules have shown wide variety of biological effects, such as antibacterial and antifungical, antileishmanial and antitrypanosomal, cardiotonic, diuretic, antiproliferative and cytotoxic [12-16]. Effects on the central and peripheral nervous system were also related. Symptoms like salivation, hallucination and seizures were observed in cases of ingestion of the poison [9]. Poisoned dogs showed mydriasis, nystagmus and opisthotonus [17, 18]. Some studies suggest mechanisms of interaction between bufadienolides and the neuromuscular junction [19, 20], causing blockage of synaptic transmission [17, 21]. Molecules such as resibufogenin and cinobufagin demonstrated actions on voltage-gated potassium channels and resibufogenin also in the voltage-gated sodium channels [22, 23].

The classical mechanism of the cardiotonic steroid molecules is to bind on the extracellular surface of the enzyme Na^+^/K^+^-ATPase, inhibiting its functioning [24, 25]. This action may also influence the performance of the nervous system [26]. On the other hand, it has demonstrated that the specificity of bufadienolides for the Na^+^/K^+^-ATPase in neurons is lower than in cardiac cells, suggesting that neurotoxicity may not be connected to it [27].

Na^+^/ K^+^-ATPase is an abundant protein in central nervous system cells, and the evaluation of its activity can help in the elucidation of neurotoxic activities [28].

One of the most common neurological diseases existing in the world population is epilepsy, and most of the patients who present this pathology do not respond to drug treatments [29, 30]. Epilepsy refers to any type of disease characterized by the occurrence of spontaneous and recurrent seizures, caused by paroxysmal discharges of brain neurons, affecting about 700.000 people in the United States and around 5 million people worldwide, representing approximately 1% of the world's population. It is believed that dysfunctions in the chemical balance of neurotransmitters may be the main cause of both development and maintenance of epileptiform electrical activity [30-33].

The study of new promising compounds for the treatment of epilepsy is extremely important, since 30% to 40% of patients with epilepsy disease have seizures relapses during the treatment and do not respond to current antiepileptic drugs [34].

Epilepsy is a large limiting factor in people's social lives, having a huge impact on the health care system and work productivity [31, 35]. Therefore, studies that search for new molecules that can act at synapses and that present potential medical use in humans are relevant. Poisons can be considered rich natural sources of bioactive molecules [36-38]. In this context, this work evaluated the neuroprotective potential of LMWF from *Rhinella scheneideri* poison, treating seizures induced by PTZ and NMDA. 

## Material and Methods

The handling of experimental animals was performed according to the Principles Ethical in Animal Experimentation (Brazilian College of Animal Experimentation [39], the Guiding Principles for Research Involving Animals and Human Beings - American Physiology Society and Ethical Guidelines for Investigations of Experimental Pain in Conscious Animals [37]. The Ethics Committee on Animal Use (CEUA) of University of São Paulo - Campus of Ribeirão Preto (Protocol 09.1.148.53.9) approved this study.

### *Rhinella schneideri* poison and low molecular weight fraction (LMWF) 

The *Rhinella schneideri* toad poison was collected by pressuring their parotoid glands of adult, male and female toads, from the animal facility of the University of São Paulo in Ribeirão Preto, accredited by Brazilian Institute of Environment and Renewable Natural Resources (IBAMA), under register number 1506748, for scientific purposes. Animals were previously cleaned and the poison dried and immediately stored at -20 °C. The dried poison (400 mg) suspended in 30 mL of MiliQ® water and the suspension was subjected to dialysis using Fisherbrand® 6000-8000 MWCO membranes. Four water changes were carried out in periods of six hours. The four waters changes containing the low molecular mass molecules that permeate the dialysis membrane were collected, frozen and lyophilized, resulting in the sample used in the assays, named the low molecular weight fraction (LMWF).

### Inhibition of Na^+^/K^+^-ATPase enzyme assays

Na^+^/K^+^-ATPase enzyme sample was obtained and purified as described by Yoneda, [40]. The inhibition of the enzymatic activity of Na^+^/K^+^-ATPase (ATPase activity) was assayed discontinuously for 30 minutes at 37 °C in a final volume of 1.0 mL. Standard assay conditions were 50 mM HEPES buffer, pH 7.5, containing 3 mM ATP, 10 mM KCl, 5 mM MgCl_2_, and 50 mM NaCl with 4 different concentrations (1.0, 10.0, 50.0 and 100.0 µg/mL) of LMWF. The reaction was initiated by the addition of 30 μL of the enzyme and it was interrupted with 0.5 mL of cold 30% trichloroacetic acid (TCA). Samples were centrifuged at 4000 *g* and 500 μL were taken from supernatant to quantify the phosphate released from ATP hydrolysis. The quantification was performed according to Heinonen and Lathi, [39], which is a colorimetric method. One volume of ammonium molybdate solution (10 mM), containing H_2_SO_4_ (5N) was added to the sample, after that, 2 volumes of acetone, and finally 1 volume of citric acid (0,4M). Each addition was followed by vortex and the absorbance of the yellow solution measured at 355 nm. The amount of released phosphate was quantified comparing with a curve standardized previously in which known amounts of phosphate were dosed following the same procedure. The measurements were performed in triplicate. Control without enzyme (negative control), to eliminate background influence on results, and control without LMWF (positive control, 100 % ATPase activity) included in each experiment.

### Anticonvulsant activity in pentylenetetrazole (PTZ) or N-methyl-D-aspartate (NMDA)-induced acute seizure models

Wistar male rats (200 to 250 g) were purchased from the Central Animal Facility of the University of São Paulo, Ribeirão Preto Campus. Animals were kept in pairs in wire-mesh cages in a room with a 12-h dark/light cycle (lights on at 7:00 a.m.). Food and water were offered *ad libitum.* After a period of two days of habituation, animals were injected with atropine sulfate (0.5 mg/kg, i.p.) and anesthetized with ketamine (80 mg/kg) combined with xylazine (10 mg/kg). Then, they were positioned on a stereotaxic Stoelting-Standard®. Local injection of lidocaine (2%) was performed and a 10 mm cannula was implanted AP - 0.9 mm, ML - 1.6 mm, DV - 3.4 mm, based on Bregma, according to the atlas of Paxinos & Watson [41]. After implantation, the cannula was fixed with dental acrylate. After the surgery, the animals received prophylactic antibiotic against infections (pentabiotic 50 mg/kg, i.p.). Animals were allowed to rest for 5-7 days to recover from the surgery. After the post-surgical time, animals (n = 6 per group) were injected by i.c.v., with 1.0 μL of different dilutions of the LMWF (0.25 to 4 μg/μL) or vehicle. After 15 min, each group received an injection of NMDA (0.17 μg/μL, i.c.v.) or PTZ (85 mg/kg, i.p., 0.1 mL). The behavior of all groups was videotaped for 30 min to assess seizure score according to Racine index [42].

### Assessment of LMWF activity on the uptake of the excitatory amino acid L-Glutamate in brain-cortical synaptosomes of Wistar rats

Cerebral cortices of male Wistar rats (200 to 250 g) were rapidly removed and homogenized on ice with 0.32 M sucrose using Potter-Elhvejen Labo Stirrer LS-50 (Yamato, USA) type equipment. The sample was centrifuged for 10 min at 1700 x g (4 °C) and the supernatant centrifuged for 20 min at 21200 x g (4 °C). The pellet was resuspended in Krebs-phosphate buffer (in mM: 124 NaCl, 5 KCl, 1.2 KH_2_ PO_4_, 0.75 CaCl_2_, 1.2 MgSO_4_, 20 Na_2_ HPO_4_, 10 glucose, pH 7.4), and used in the assay of [^3^H]-L*-*Glutamate uptake. The protein content was determined by Lowry et al. [43], modified by Hartree, [44]. The synaptosomes were resuspended in Krebs-phosphate buffer and preincubated for 5 min at 37 °C in the presence or absence of different concentrations of LMWF (10^-5^ to 10 µg/µL). The uptake experiment was initiated by addition of [^3^H]-L*-*Glutamate (36 nM, final concentration) to the synaptosomes suspension (100 μg protein/mL) and incubated for 3 minutes at 37 °C. The reaction was finished by centrifugation at 4 °C. Aliquots of the supernatant were transferred to scintillation tubes containing 5 mL of water-miscible biodegradable scintillation liquid (ScintiVerse, Fisher Scientific, USA), and their radioactivities were quantified by a liquid scintillation spectrophotometer (Beckman, model LS-6800) with 2% error and counting efficiency for 3H^+^ of 50% [45]

## Results

### Na^+^/K^+^-ATPase inhibition assays

**Figure 1.**
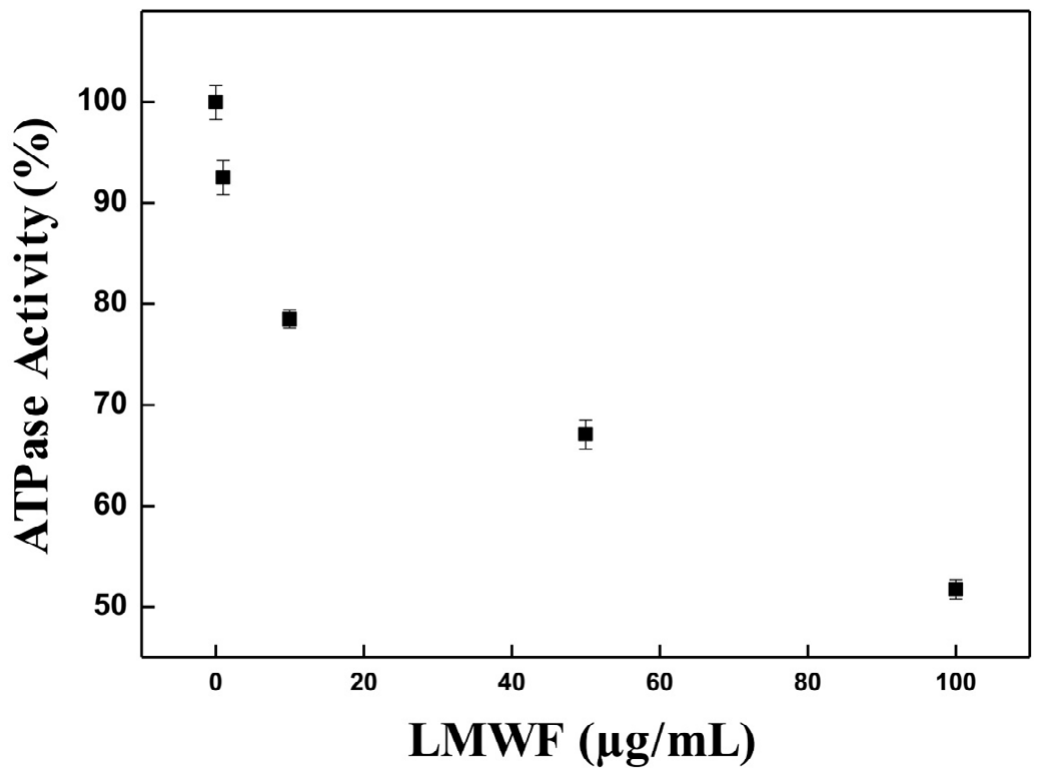
**Inhibition Assays of Na^+^/K^+^-ATPase Enzyme**. Enzymatic inhibition activity induced by LMWF (1; 10; 50 and 100 μg/mL) had demonstrated a dose dependent concentration and the IC_50_ was 107.5 µg/mL. Control without enzyme (negative control) and control without LMWF (positive control, 100 % ATPase activity in figure) included in each experiment. The assay was performed in triplicate.

The assay performed with Na^+^/K^+^-ATPase enzyme isolated from the membrane has shown a dose-dependent inhibition. The IC 50%, the concentration that caused 50% of inhibition, determined by the fit of the graph (exponential decay), was 107.5 μg/mL (Fig. 1). This fraction probably contains the bufadienolides, considered mainly responsible for the inhibitory action of the enzyme.

### LMWF anticonvulsant activity in PTZ-induced seizure model

**Figure 2.**
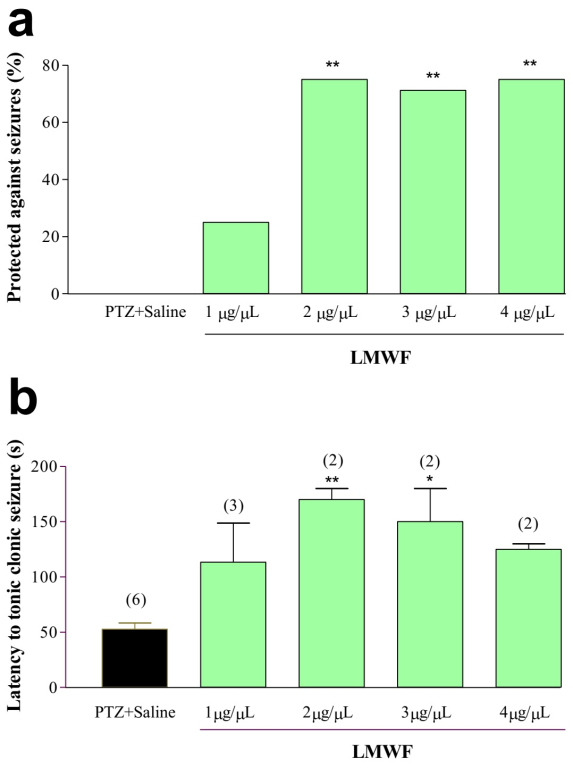
**LMWF anticonvulsant activity in PTZ-induced seizure model**. **A**: Percentages of animals protected against PTZ-induced seizures after injection of different concentrations of LMWF. The frequencies of protected animals were analyzed using the chi-square test, followed by Fischer's test. *p <0.05 . ** p <0.01 compared to the control. **B**: Mean ± SEM of the latencies for triggering seizures in animals not protected against PTZ-induced seizures after injection of different concentrations of LMWF. Data were analyzed using one-way ANOVA followed by the Tukey post-test. *p <0.05 and **p <0.01 compared to the control. The numbers in parentheses above the columns represent the number of animals used.

Pretreatment with different concentrations of LMWF inhibited tonic-clonic seizures induced by systemic injection of PTZ in a dose-dependent manner. Treatment with LMWF at doses of 2, 3 and 4 μg/μL significantly protected animals from PTZ-induced seizures (χ 2 = 10.26, 4, p = 0.0362) (Fig. 2A). Also, animals treated with LMWF at concentrations of 2 and 3 μg/μL that developed tonic-clonic seizures presented a higher latency than the animals in the control group [F (4.5) = 7.74; p = 0.0032] (Fig. 2B).

### LMWF anticonvulsant activity in NMDA-induced seizure model

**Figure 3.**
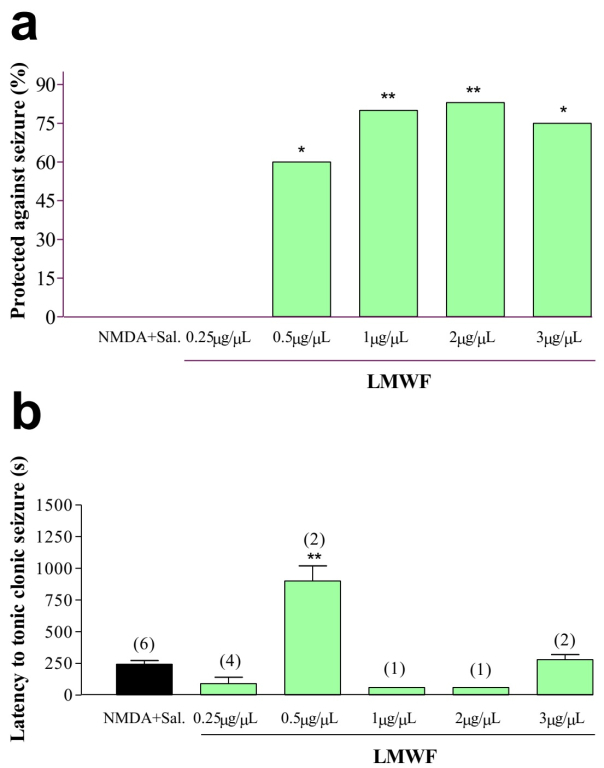
**LMWF anticonvulsant activity in NMDA-induced seizure model**. **A**: Mean percentages of animals protected against NMDA-induced seizures after injection of different concentrations of LMWF. Data analyzed using the chi-square test followed by Fischer's test. * p <0.05 and ** p <0.01 compared to the control. **B**: Mean ± SEM of the latencies for triggering seizures in animals not protected against NMDA-induced seizures after injection of different concentrations of LMWF. Data were analyzed using ANOVA test followed by Tukey post-test. * p <0.05 and ** p <0.01. The numbers in parentheses above the columns represent the number of animals used.

In NMDA-induced seizure experiment the frequency of animals protected against seizures was significantly higher in animals treated with LMWF doses of 0.5, 1, 2 and 3 μg/μL (χ 2 = 15.67, 5, p = 0.0079) (Fig. 3A). Moreover, animals that received the concentration of 0.5 μg/μL and developed seizures presented a significantly higher latency to tonic-clonic behavior compared to control group [F (4.5) = 7.74; p = 0.0032] (Fig. 3B).

### Assessment of LMWF activity on the uptake of the excitatory amino acid [3H]-L-Glutamate in brain-cortical synaptosomes of Wistar rats

**Figure 4.**
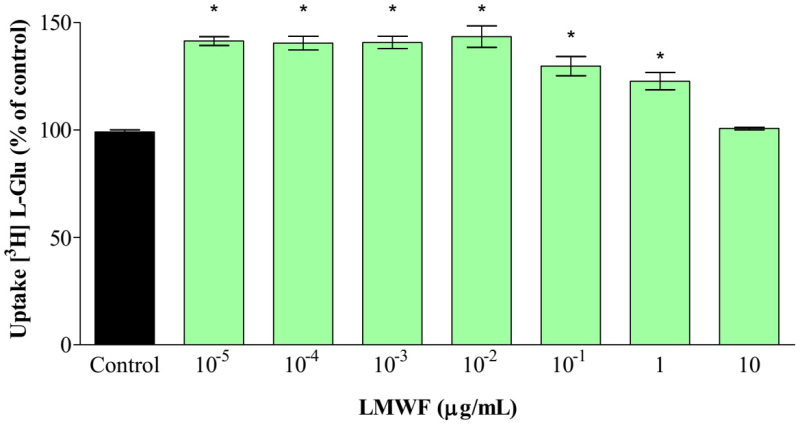
**Assessment of [3H]-L-GLU uptake induced by LMWF into synaptosomes**. Effects of increasing concentrations of LMWF (10^-5^ to 10 μg/μL) on [3H]-L-Glu uptake. Data presented as mean ± SEM of three experiments performed in triplicate. Statistical significance was determined by the Newman Keuls post-test. * p <0.001 compared to the control.

LMWF, which probably contains bufadienolides, induces increased uptake of the amino acid [^3^H]-L*-*Glutamate in brain-cortical synaptosomes of Wistar rats. This increase in uptake was observed mainly at the lowest concentrations tested (10^-5^ to 10^-2^) (Fig. 4). However, it has observed that at higher concentrations (10 µg/mL) this effect is reversed, reaching values equal to those of the control. This unexpected effect may be a consequence of the presence of components with antagonistic actions present in LMWF.

## Discussion

The results showed that when exposed to different concentrations of LMWF, the Na^+^/K^+^-ATPase enzyme had its activity inhibited in a dose-dependent manner. An IC_50%_ of 107.5 μg/mL obtained for LMWF under the assay conditions. This is an important result for pharmacological and toxicological evaluation of the sample.

The Na^+^/K^+^-ATPase, or Na^+^/K^+^ pump, is an enzyme located in the plasma membrane of a large part of the eukaryotic cells and carries the Na^+^ and K^+^ ions against their electrochemical gradients, presenting a vital role for cellular homeostasis. It is also responsible for initiating cellular signalling processes that involve reactive oxygen species, important in several pathologies. The inhibition and the onset of the signalling function of Na^+^/K^+^-ATPase caused by the interaction with cardiotonic glycosides which bind on the α-subunit of the enzyme classically known [28, 46]. In addition, it is also known and documented that different components from frog poisons have antineoplastic activities and actions on the central nervous system due to their effects on Na^+^/K^+^-ATPase [7].

Na^+^/K^+^-ATPase inhibitors present a high binding affinity that can modify according to the type of structure that these molecules have. The bonds can be stronger or weaker depending on the steroid nucleus or the lactone [47]. The LMWF is composed of a great variety of molecules and, among them, those responsible for the inhibition of this enzyme.

The results also demonstrated the suppression of generalized tonic-clonic seizures induced by PTZ or NMDA. In assays that LMWF was not able to inhibit seizures, it increased the latency time to trigger the symptoms. However, when the dose increased, it was observed a reduction in the percentage of protected animals and a decrease in the latency time for the onset of seizures. The results indicated that the toxins present in LMWF differed in relation to the effect induced in the central nervous system (CNS). Probably, some of them have depressant effects and others excitatory effects. Depending on the concentration of the fraction one or the other action becomes more effective.

Some works described that extracts of plants of the genus *Kalanchoe* (Crassulaceae) that contains bufadienolides, inhibit Na^+^ /K^+^-ATPase activity, cause seizures and show depressant actions in CNS. Oleandrin, a plant cardenolide, was also described as neuroprotective [48-51]. These effects are similar to those observed with LMWF.

Considering that LMWF increases the uptake of the excitatory amino acid L-Glutamate in brain-cortical synaptosomes of Wistar rats, this action can be related to the protection against seizure effect observed in the seizure assays.

Neuronal damages including cell death can be avoided by performing the glutamate decrease in the synaptic cleft, and the uptake carried out by these transporters is the main mechanism for the end of excitatory neurotransmission [52, 53].

Since the classic action of bufadienolides in the inhibition of Na^+^/K^+^-ATPase enzyme, and considering that they are essential for the functionality of central nervous system cells, it is certain that these toxins will have actions on this system [7, 28]. This inhibition affects the flow of ions, causing an influence on the transport of glutamate. The extracellular concentration of glutamate is controlled by a family of sodium-dependent carrier proteins, the excitatory amino acid transporters (EAATs), which are divided into 5 structurally distinct subtypes (EAATs 1-5) and they are directly implicated in several pathologies such as epilepsy, Alzheimer's, cerebral ischemia, among others. Some of the carriers are directed by a gradient of sodium and potassium [54, 55].

These results strongly suggest that there are molecules in the LMWF that have anticonvulsive potential and can be an interesting tool in the study and prospection of new anticonvulsant drugs. Additionally, these results are unprecedented and open perspectives for the development of new researches, aiming the characterization of the neuroprotective mechanism of LMWF components.

## Conclusion

The results show that LMWF was able to inhibit convulsive seizures induced by PTZ and NMDA and when it was not able to inhibit the seizures, it increased the seizure latency time. LMWF was also able to inhibit the Na^+^/K^+^-ATPase and increase the levels of [^3^H]-L*-*glutamate uptake. LMWF is a rich source of components with biological functions of high medical and scientific interest. It has molecules that explore the central nervous system, triggering positive responses in relation to the assays performed. However, other studies with isolated molecules should be performed to assess their pharmacological potential.

## Abbreviations

 LMWF: Low Molecular Weight Fraction; L-Glu: L-Glutamate; PTZ: pentylenetetrazole; NMDA: N-methyl-D-aspartate; COBEA: Brazilian College of Animal Experimentation; CEUA: Ethics Committee on Animal Use; MWCO: Molecular weight cut-off.
